# The Clinical Characteristics of ARDS in Children With Hematological Neoplasms

**DOI:** 10.3389/fped.2021.696594

**Published:** 2021-07-08

**Authors:** Qiao Zhang, Wen-ting Hu, Fan Yin, Han Qian, Ying Wang, Bi-ru Li, Juan Qian, Yan-jing Tang, Bo-tao Ning

**Affiliations:** ^1^Department of Intensive Care Medicine, Shanghai Children's Medical Center, Shanghai Jiaotong University School of Medicine, Shanghai, China; ^2^Department of Hematology and Oncology, Shanghai Children's Medical Center, Shanghai Jiaotong University School of Medicine, Shanghai, China; ^3^Shanghai Engineering Research Center of Intelligence Pediatrics (SERCIP), Shanghai, China

**Keywords:** children, hematological neoplasms, acute respiratory distress syndrome, agranulocytosis, clinical characteristics

## Abstract

In order to explore the clinical characteristics of pediatric patients admitted to the pediatric intensive care unit (PICU) who suffered from hematological neoplasms complicated with acute respiratory distress syndrome (ARDS), we retrospectively analyzed 45 ARDS children with hematological neoplasms who were admitted to the PICU of Shanghai Children's Medical Center from January 1, 2014, to December 31, 2020. The 45 children were divided into a survival group and a non-survival group, a pulmonary ARDS group and an exogenous pulmonary ARDS group, and an agranulocytosis group and a non-agranulocytosis group, for statistical analysis. The main clinical manifestations were fever, cough, progressive dyspnea, and hypoxemia; 55.6% (25/45) of the children had multiple organ dysfunction syndrome (MODS). The overall mortality rate was 55.6% (25/45). The vasoactive inotropic score (VIS), pediatric critical illness scoring (PCIS), average fluid volume in the first 3 days and the first 7 days, and the incidence of MODS in the non-survival group were all significantly higher than those in the survival group (*P* < 0.05). However, total length of mechanical ventilation and length of hospital stay and PICU days in the non-survival group were significantly lower than those in the survival group (*P* < 0.05). The PCIS (OR = 0.832, *P* = 0.004) and the average fluid volume in the first 3 days (OR = 1.092, *P* = 0.025) were independent risk factors for predicting death. Children with exogenous pulmonary ARDS were more likely to have MODS than pulmonary ARDS (*P* < 0.05). The mean values of VIS, C-reactive protein (CRP), and procalcitonin (PCT) in children with exogenous pulmonary ARDS were also higher (*P* < 0.05). After multivariate analysis, PCT was independently related to exogenous pulmonary ARDS. The total length of hospital stay, peak inspiratory pressure (PIP), VIS, CRP, and PCT in the agranulocytosis group were significantly higher than those in the non-agranulocytosis group (*P* < 0.05). Last, CRP and PIP were independently related to agranulocytosis. In conclusion, children with hematological neoplasms complicated with ARDS had a high overall mortality and poor prognosis. Children complicated with MODS, positive fluid balance, and high VIS and PCIS scores were positively correlated with mortality. In particular, PCIS score and average fluid volume in the first 3 days were independent risk factors for predicting death. Children with exogenous pulmonary ARDS and children with agranulocytosis were in a severely infected status and more critically ill.

## Introduction

Due to repeated chemotherapy and infiltration of malignant cells, children with hematological neoplasms often evolve into having severe bone marrow suppression and then become prone to a series of opportunistic infections, which are one of the foremost factors why children with hematologic neoplasms exhibit complications including acute respiratory distress syndrome (ARDS). ARDS is a sign of systematic inflammatory response syndrome (SIRS) in the lungs, caused by various direct and indirect injuries, characterized by the destruction of the integrity of the alveolar–capillary barrier and increased permeability, leading to the accumulation of large amounts of protein-rich fluid in the alveoli and interstitial lung, which, in turn, leads to the formation of a hyaline membrane in the alveoli (1). The whole process seriously interferes with gas exchange, resulting in progressive dyspnea and intractable hypoxemia, accompanied with high mortality rate. Generally speaking, the mortality rate of children with ARDS without underlying diseases is about 30% (2). However, the mortality rate among patients with hematological neoplasms complicated with ARDS has been reported to be as high as 56–77% (3–5), and the prognosis is different due to the etiology and individual differences. A considerable amount of literature has been published describing adult patients with hematological neoplasms complicated with ARDS, whereas very little was found on the question of children with ARDS, so by analyzing the clinical characteristics of the whole group and each subgroup, we hope to describe the clinical characteristics, outcome, and predictors of poor outcome in children with hematological neoplasms and ARDS.

## Materials and Methods

### Case Data and Grouping

Clinical data of the total 45 children diagnosed with hematological neoplasms complicated with ARDS were collected from January 2014 to December 2020 in the Pediatric Intensive Care Unit of Shanghai Children's Medical Center, which is affiliated with Shanghai Jiao Tong University School of Medicine. All the children included met the diagnostic criteria for pediatric ARDS set out in the 2015 PALICC meeting (6). According to clinical outcome, etiology of ARDS, and absolute count of peripheral granulocytes, the children were divided into a survival group and a non-survival group (20/25), a pulmonary ARDS group and an exogenous pulmonary ARDS group (34/11), and an agranulocytosis group (absolute value of granulocytes < 0.5 × 10^9^/L) and a non-agranulocytosis group (18/27).

This study was approved by the Ethics Committee of Shanghai Children's Medical Center (SCMCIRB-W2021023).

### Data Collection

Use the electronic medical record system (EMR) to analyze the following data examined with a diagnosis of ARDS: age, gender, white blood cell count, neutrophil, C-reactive protein (CRP), procalcitonin (PCT) level, respiratory rate, positive end expiratory pressure (PEEP), peak inspiratory pressure (PIP), oxygenation index (OI), duration of mechanical ventilation, vasoactive inotropic score (VIS), pediatric critical illness score (PCIS), fluid balance status in the first 3 days and 7 days after the diagnosis of ARDS, continuous renal replacement therapy (CRRT) status, total length of hospital stay, length of PICU stay, primary existing blood diseases, and co-existing multiple organ dysfunction syndrome (MODS).

### Therapy

Refer to the treatment guideline of the 2015 PALICC Pediatric Acute Respiratory Distress Syndrome Consensus Conference (6), and provide corresponding ventilatory support to the children. Some children received blood purification due to the obvious SIRS. According to monitoring of bedside cardiopulmonary ultrasound, appropriate fluid resuscitation, and vasoactive drugs are applied to children with unstable hemodynamics. Other treatments include standard supportive treatment such as antibiotics and blood transfusion.

### Statistical Analysis

All statistical analysis in this study was performed using SPSS24.0 statistical software. Measurement data were expressed as mean ± standard deviation (x ± s), and median (25% percentile, 75% percentile) was used for skew distribution. χ^2^ test or Fisher's exact test was used to compare the categorical variables, while the Student's *t*-test or the Mann–Whitney *U*-test was used to compare the continuous variables. Univariate and multivariate logistic regression analyses were used in the assessment of risk factors for mortality. *P* < 0.05 was considered to be statistically significant.

### Definition

According to the oxygenation index (OI) to define the severity of ARDS, OI = MAP × FiO_2_ × 100/PaO_2_ (7). Agranulocytosis is defined as the absolute value of neutrophils < 0.5 × 10^9^, and the blood routine data were within 3 days after the diagnosis of ARDS; according to detection method of our hospital, an increase in CRP is defined as >8 mg/L, and the elevation of PCT is defined as >0.5 ng/ml. Considering the VIS, we select the maximum value within 3 days after the diagnosis of ARDS; the specific formula is (8): VIS = 100 × norepinephrine (μg/kg/min) + 100 × adrenaline (μg/kg/min) + 10 × Milrinone (μg/kg/min) + 1 × Dobutamine (μg/kg/min) + 1 × Dopamine (μg/kg/min); the method for judging the liquid balance of the first 3 and 7 days is as follows: the total input in the first 3 or 7 days minus the total output (a positive number indicates a positive balance, a negative number indicates a negative balance, unit: ml), then as an integer, divided by the corresponding number of days and the weight of the child. The final result is expressed in ±ml/kg/day.

## Results

### General Situation

During the 7-year study period, 2,792 children with hematological neoplasms were admitted to our hospital. Only 45 children with hematological neoplasms were included for this analysis. The prevalence of ARDS in children with hematological neoplasms was 1.61% (45/2,792). There are 26 males and 19 females in our study. The ratio of male to female was 1.4:1, and the median age of onset of ARDS was 4 years (1.92, 6). Among them, there were 21 cases of acute lymphocytic leukemia (46.7%), 17 cases of acute myeloid leukemia (37.8%), 1 case of biclonal leukemia (2.2%), 2 cases of myelodysplastic syndrome (4.4%), 1 case of Hodgkin lymphoma (2.2%), and 3 cases (6.7%) of non-Hodgkin's lymphoma.

### Clinical Characteristics

All children exhibited clinical manifestations of fever, cough, and progressive dyspnea during the course of their illness. There were 34 cases (75.6%) of pulmonary ARDS, 11 cases (24.4%) of exogenous pulmonary ARDS; 18 cases (40%) manifested agranulocytosis at the time of diagnosis of ARDS, while the number of non-agranulocytosis was 27 (60%); 25 cases (55.6%) had multiple organ dysfunction, among them, liver and kidney dysfunction were the most common; 10 cases had 2 organ dysfunction (22.2%), and 15 cases (33.3%) were combined with three or more organ dysfunction.

There were 42 cases (93.3%) with elevated CRP, with an average of 98.3 ± 63.4 mg/L, and 36 cases (80%) with elevated PCT, with a median of 2.8 (1.2, 13.2) ng/ml. There were 17 cases (37.8%) with positive culture of body fluids, including sputum culture, blood culture, urine culture, etc. Among them, the positive rate of sputum culture was the highest, accounting for 82.4% (14/17). The percentage rate of *Acinetobacter baumannii* infections was the highest (9 cases, 52.9%), followed by *Pseudomonas aeruginosa* (2 cases, 11.8%), and *Pneumocystis carinii* (2 cases, 11.8%); G/GM test had 9 positive cases (20%).

The majority of the children (93.3%, *n* = 42) had moderate-to-severe ARDS (moderate in 48.9%, *n* = 22; and severe in 44.4%, *n* = 20). There was no significant correlation between ARDS severity and mortality (*P* = 0.404).

In the first 3 days, there were 24 children positively balanced (53.3%), and 21 cases (46.7%) were negatively balanced. However, among the first 7 days, 26 cases were positively balanced (57.8%), and the number of negative fluid balance is 19 (42.2%); for more details, see Table 1.

**Table 1:** Fluid balance status in the first 3 and 7 days.

**Day outcome**	**Day 3 (*****n*** **=** **45)**		**Day 7 (*****n*** **=** **45)**	
	**Positive**	**Negative**	**Positive**	**Negative**
	**(*n* = 24)**	**(*n* = 21)**	**(*n* = 26)**	**(*n* = 19)**
Survivor	7 (29.2%)	13 (61.9%)	9 (34.6%)	11 (57.9%)
Non-survivor	17 (70.8%)	8 (38.1%)	17 (65.4%)	8 (42.1%)

The mean length of hospital stay was 28.6 ± 19.5 days, and the average length of stay in the PICU was 14.1 ± 11.3 days.

### Treatment and Outcome

All the children in this study were treated with targeted therapeutic intervention; for example, due to fever and immunosuppressed state, all children received broad-spectrum antibiotics. Simultaneously, all have experienced a conventional ventilator during the course of disease, of which 11 cases (24.4%) were treated with a high-frequency ventilator to optimize the assisted ventilation strategy. A total of 12 cases (26.7%) were subjected to prone positioning, and 8 of the children who received prone positioning died (8/12, 66.7%). Compared with children who did not receive prone positioning, the mortality rate of children who received prone positioning was higher (66.7% vs. 51.5%, *P* = 0.366 > 0.05), but there was no statistical significance. All the children's ventilator settings were as follows: the average PIP was 28.5 ± 5.4 cm H_2_O; the average PEEP was 10.4 ± 2.9 cm H_2_O; the average FiO_2_ was 0.7 ± 0.2; OI is 18.0 ± 10.2.

The score of vasoactive drugs was evaluated in 45 children, and the median score is 12.5 (0, 60); the scores of 31 children (68.9%) were <50, including 14 children (31.1%) who had never used vasoactive drugs. There were eight children (17.8%) with scores ranging from 50 to 100, and six cases (13.3%) with scores >100. All the six cases with scores >100 eventually died.

Due to acute kidney injury (AKI), eight children (17.8%) received CRRT therapy, with a median treatment time of 1.5 ± 1.1 days. One child survived and the remaining seven died, with a higher mortality rate (87.5 vs. 48.6%) in children treated with CRRT compared to those not treated with CRRT (*P* < 0.05).

### Subgroup Analysis

#### The Survival Group and the Non-Survival Group

The average hospital stay of the living group (20 cases) and the death group (25 cases) were 41.2 ± 19.7 and 18.6 ± 13.2 days, respectively. The mean PICU stay time was 21.6 ± 12.8 and 8.2 ± 5.6 days, respectively, and there was statistical difference between the two groups (*P* < 0.05). The average mechanical ventilation time of the survival group and the non-survival group was 17.4 ± 12.6 and 8.2 ± 5.7 days, respectively, with a significant difference between groups (*P* < 0.05). The ratio of high scores of vasoactive drugs (>50) in the survival group was lower than that in the non-survival group (*P* < 0.05), and the mean VIS score of non-survival group was 30 (10, 97.5), while the average VIS score of the survival group was 2.5 (0, 17.3). There was a statistical difference between the two groups (*P* = 0.002). The difference of PCIS between the survival and non-survival group was 79.1 ± 5.3 and 66.6 ± 8.3, respectively (*P* < 0.001). The average positive fluid balance in the first 3 and 7 days was significantly higher among non-survivors than survivors [4.5 (−3.4, 10.3) vs. −2.7 (−12.4, 5.4) ml/kg/day, *P* = 0.019; 3.9 (−3.9, 13.8) vs. −1.1 (−9.7, 3.9) ml/kg/day, *P* = 0.029, respectively].

MODS occurred in 18 children in the non-survival group (18/25, 72%), and the incidence of MODS in the non-survival group was significantly higher than that in the survival group (7/20, 35.0%) (*P* = 0.013). Of the eight children treated with CRRT therapy, seven died and one survived (see Table 2).

**Table 2:** The characteristics of ARDS in children with hematologic neoplasms: survivors and non-survivors.

**Characteristic**	**Survivor (*n* = 20)**	**Non-survivor (*n* = 25)**	***P*-value**
Sex (male/female)	13/7	13/12	0.380
Age (*n* %)			0.415
<6 month	2 (10.0%)	0 (0%)	
<2 year	3 (15.0%)	7 (28.0%)	
2–6 year	10 (50.0%)	12 (48.0%)	
7–17 year	5 (25.0%)	6 (24.0%)	
Agranulocytosis (*n* %)	8 (40.0%)	10 (40.0%)	1.000
CRP ($\bar{x}\;\pm$ s, mg/L)	86.2 ± 63.2	112.8 ± 65.4	0.175
PCT (*n*, ng/mL)	1.2 (0.4, 3.3)	2.2 (1.1, 14.2)	0.138
VIS (*n* %)			0.037
<50	17 (85.0%)	14 (56.0%)	
50–100	3 (15.0%)	5 (20.0%)	
>100	0 (0%)	6 (24.0%)	
VIS (*n*)	2.5 (0, 17.3)	30 (10, 97.5)	0.002
Fluid balance in the first 3 days (ml/kg/d)	−2.7 (−12.4, 5.4)	4.5 (−3.4, 10.3)	0.019
Fluid balance in the first 7 days (ml/kg/d)	−1.1 (−9.7, 3.9)	3.9 (−3.9, 13.8)	0.029
Total length of mechanical ventilation [($\bar{x}\;\pm$ s), d]	17.4 ± 12.6	8.2 ± 5.7	0.007
PEEP [($\bar{x}\;\pm$ s), cmH_2_O]	9.9 ± 2.7	10.5 ± 3.2	0.818
PIP [($\bar{x}\;\pm$ s), cmH_2_O]	27.6 ± 5.3	29.8 ± 4.9	0.147
OI($\bar{x}\;\pm$ s)	16.2 ± 7.4	19.5 ± 12.0	0.404
CRRT therapy (*n* %)			0.045
Yes	1 (5.0%)	7 (28.0%)	
No	19 (95.0%)	18 (72.0%)	
Total hospital stay [($\bar{x}\;\pm$ s), d]	41.2 ± 19.7	18.6 ± 13.2	0.042
Total length of PICU [($\bar{x}\;\pm$ s), d]	21.6 ± 12.8	8.2 ± 5.6	0.000
Type of primary blood tumors [*n* (%)]			0.835
Acute lymphocytic leukemia	9 (45.0%)	12 (48.0%)	
Acute myeloid leukemia	9 (45.0%)	8 (32.0%)	
Biclonal leukemia	0 (0%)	1 (4.0%)	
Others^*^	2 (10.0%)	4 (16.0%)	
MODS [*n* (%)]			0.013
With MODS	7 (35.0%)	18 (72.0%)	
Without MODS	13 (65.0%)	7 (28.0%)	
PCIS score($\bar{x}\;\pm$ s)	79.1 ± 5.3	66.6 ± 8.3	0.000
Severity of ARDS [*n* (%)]			0.637
Mild	1 (5.0%)	0 (0%)	
Moderate	11 (55.0%)	16 (64.0%)	
Severe	8 (40.0%)	9 (36.0%)	

*CRP, C Reactive Protein; PCT, Procalcitonin; VIS, vasoactive inotropic score; PEEP, Positive end-expiratory pressure; PIP, Peak Inspiratory Pressure; OI, Oxygenation index; CRRT, continuous renal replacement therapy; MODS, multiple organ dysfunction syndrome; PCIS, pediatric critical illness scoring; ARDS, acute respiratory distress syndrome.*

* *Others included biclonal leukemia (n = 1), myelodysplastic syndrome (n = 2), Hodgkin lymphoma (n = 1), and non-Hodgkin's lymphoma (n = 3)*.

#### Pulmonary ARDS and Exogenous Pulmonary ARDS

The mortality rates of children with pulmonary ARDS and exogenous pulmonary ARDS were 52.9% (18/34) and 63.6% (7/11), respectively, with no statistical difference between the two groups (*P* > 0.05). There were statistical differences in CRP, PCT, VIS, and MODS between the two groups, among which CRP and PCT in the exogenous pulmonary ARDS group were significantly higher than those in the pulmonary ARDS group [142.1 ± 67.7 vs. 87.7 ± 59.4, *P* = 0.021; 11.7 (3.2, 28.4) vs. 1.2 (0.5, 3.7), *P* = 0.003]. The VIS of the exogenous pulmonary ARDS group was significantly higher than that of the pulmonary ARDS group [85 (30, 138.8) vs. 10 (0, 30.6), *P* = 0.001]. The incidence of MODS in children with pulmonary ARDS and exogenous pulmonary ARDS was 44.1% (15/34) and 81.8% (9/11), respectively, and there was a significant statistical difference between the two groups (*P* = 0.029); that is, children with exogenous pulmonary ARDS were more likely to be complicated with MODS. As for fluid balance status, the average positive fluid balance status in the first 3 and 7 days between exogenous pulmonary ARDS and pulmonary ARDS was as follows: 4 (−4.1, 15.4) ml/kg/day vs. −0.5 (−5.4, 8.2) ml/kg/day, *P* = 0.410; 4.2 (1.9, 5.3) vs. −0.3 (−10.1, 5.5) ml/kg/day, *P* = 0.058, respectively (see Table 3 for details).

**Table 3:** The characteristics of ARDS in children with hematologic neoplasms: pulmonary ARDS and exogenous pulmonary ARDS.

	**Pulmonary ARDS (*n* = 34)**	**Exogenous pulmonary ARDS (*n* = 11)**	***P*-value**
Clinical outcome (*n* %)			0.535
Survivor	16 (47.0%)	4 (36.3%)	
Non-survivor	18 (53.0%)	7 (63.6%)	
Sex (male/female)	20/14	6/5	1.000
Age (*n* %)			0.424
<6 months	1 (2.9%)	1 (9.1%)	
<2 years	7 (20.6%)	3 (27.3%)	
2–6 years	16 (47.1%)	6 (54.5%)	
7–17 years	10 (29.4%)	1 (9.1%)	
Total length of mechanical ventilation [($\bar{x}\;\pm$ s), d]	12.5 ± 10.4	11.6 ± 10.8	0.706
Total hospital stay [($\bar{x}\;\pm$ s), d]	27.1 ± 18.9	33.3 ± 22.5	0.411
Total length of PICU [($\bar{x}\;\pm$ s), d]	14.3 ± 11.6	13.60 ± 11.7	0.845
PEEP [($\bar{x}\;\pm$ s), cmH_2_O]	10.3 ± 2.9	10.0 ± 3.5	0.540
PIP [($\bar{x}\;\pm$ s), cmH_2_O]	28.9 ± 5.5	28.5 ± 4.1	0.845
OI ($\bar{x}\;\pm$ s)	18.3 ± 10.0	17.1 ± 11.6	0.411
Fluid balance in the first 3 days (ml/kg/d)	−0.5 (−5.4, 8.2)	4 (−4.1, 15.4)	0.410
Fluid balance in the first 7 days (ml/kg/d)	−0.3 (−10.1, 5.5)	4.2 (1.9, 5.3)	0.058
CRP ($\bar{x}\;\pm$ s, mg/L)	87.7 ± 59.4	142.1 ± 67.7	0.021
PCT (*n*, ng/mL)	1.2 (0.5, 3.7)	11.7 (3.2, 28.4)	0.003
VIS [*n* (%)]			0.034
<50	26 (76.5%)	5 (45.4%)	
50–100	6 (17.6%)	2 (18.2%)	
>100	2 (5.9%)	4 (36.4%)	
VIS (*n*)	10 (0, 30.6)	85 (30, 138.8)	0.001
MODS [*n* (%)]			0.029
With MODS	15 (44.1%)	9 (81.8%)	
Without MODS	19 (55.9%)	2 (18.2%)	
ARDS [*n* (%)]			1.000
Mild	1 (2.9%)	0 (0.0%)	
Moderate	20 (58.8%)	7 (63.6%)	
Severe	13 (38.3%)	4 (36.4%)	

*PEEP, Positive end-expiratory pressure; PIP, Peak Inspiratory Pressure; OI, Oxygenation index; CRP, C Reactive Protein; PCT, Procalcitonin; VIS, vasoactive inotropic score; MODS, multiple organ dysfunction syndrome; ARDS, acute respiratory distress syndrome*.

#### The Agranulocytosis Group and the Non-agranulocytosis Group

There is no significant difference in the mortality between the agranulocytosis group and the non-agranulocytosis group (55.6 vs. 55.6%), and there is no difference in composition ratio of age, sex, PEEP, and whether or not complicated by MODS, but there are differences in total hospitalization time, VIS, PIP, CRP, and PCT values. The average hospitalization time of the two groups was 36.6 ± 21.2 and 23.3 ± 17.2 days, respectively (*P* = 0.032). The mean values of VIS were 6.3 (26.3, 105) and 10 (0, 32.5), respectively (*P* = 0.045). The mean values of PIP were 26.3 ± 3.3 and 30.5 ± 5.6, respectively (*P* = 0.018). The mean values of CRP were 139.3 ± 60.6 and 75.5 ± 55.6 mg/L, respectively (*P* = 0.001). The mean values of PCT were 3.2 (1.5, 13.9) and 1.2 (0.4, 7.3) ng/ml, respectively (*P* = 0.041) (see Table 4 for details).

**Table 4:** The characteristics of ARDS in children with hematologic neoplasms: agranulocytosis and non-agranulocytosis group.

	**Agranulocytosis (*n* = 18)**	**Non-agranulocytosis (*n* = 27)**	***P*-value**
Clinical outcome [*n* (%)]			1.000
Survivor	8 (44.4%)	12 (44.4%)	
Non-survivor	10 (55.6%)	15 (55.6%)	
Sex (male/female)	11/7	15/12	0.712
Age (*n* %)			0.744
<6 months	1 (5.6%)	1 (3.7%)	
<2 years	5 (27.8%)	4 (14.8%)	
2–6 years	8 (44.4%)	15 (55.6%)	
7–17 years	4 (22.2%)	7 (25.9%)	
Total length of mechanical ventilation [($\bar{x}\;\pm$ s), d]	13.2 ± 11.1	11.7 ± 10.1	0.798
Total hospital stay [($\bar{x}\;\pm$ s), d]	36.6 ± 21.2	23.3 ± 17.2	0.032
Total length of PICU [($\bar{x}\;\pm$ s), d]	15.2 ± 12.2	13.4 ± 11.2	0.763
PEEP [($\bar{x}\;\pm$ s), cmH_2_O]	9.8 ± 2.6	10.6 ± 3.3	0.607
PIP [($\bar{x}\;\pm$ s), cmH_2_O]	26.3 ± 3.3	30.5 ± 5.6	0.018
OI ($\bar{x}\;\pm$ s)	16.2 ± 7.8	19.3 ± 11.7	0.494
CRP ($\bar{x}\;\pm$ s, mg/L)	139.3 ± 60.6	75.5 ± 55.6	0.001
PCT (*n*, ng/mL)	3.2 (1.5, 13.9)	1.2 (0.4, 7.3)	0.041
VIS (*n* %)			0.085
<50	10 (55.6%)	21 (77.8%)	
50–100	3 (16.6%)	5 (18.5%)	
>100	5 (27.8%)	1 (3.7%)	
VIS (*n*)	6.3 (26.3, 105)	10 (0, 32.5)	0.045
CRRT therapy (*n* %)			0.694
Yes	4 (22.2%)	4 (14.8%)	
No	14 (77.8%)	23 (85.2%)	
Cause of ARDS (*n* %)			0.086
Pulmonary	11 (61.1%)	23 (85.2%)	
Exogenous pulmonary	7 (38.9%)	4 (14.8%)	
MODS (*n* %)			
With MODS	8 (44.4%)	16 (59.3%)	0.329
Without MODS	10 (55.6%)	11 (40.7%)	

*PEEP, Positive end-expiratory pressure; PIP, Peak Inspiratory Pressure; OI, Oxygenation index; CRP, C Reactive Protein; PCT, Procalcitonin; VIS, vasoactive inotropic score; CRRT, continuous renal replacement therapy; ARDS, acute respiratory distress syndrome; MODS, multiple organ dysfunction syndrome*.

### Binary Logistic Regression Analysis

After univariate analysis, multivariate analysis was performed in three subgroups. Statistically significant independent variables (VIS, PCIS, MODS, and average liquid balance in the first 3 days and the first 7 days) between the survival and the non-survival group (CRP, PCT, VIS, and MODS), between the pulmonary ARDS and the exogenous pulmonary ARDS group (CRP, PCT, VIS, and PIP), and between the agranulocytosis and non-agranulocytosis group were respectively included in the multivariate analysis. With outcomes, etiology of ARDS, and absolute count of peripheral granulocytes as dependent variables, binary logistics regression analysis was conducted, and the entire results are shown in Table 5 for details.

**Table 5:** Results of multivariate analysis among three subgroups.

**Variables**	***P*-value**	**Odds ratio**	**95% CI**
**** **Factors independently associated with hospital mortality**			
PCIS	0.004	0.832	0.734–0.943
Fluid balance in the first 3 days (ml/kg/d)	0.025	1.092	1.011–1.180
**** **Factors independently associated with etiology of ARDS**			
PCT	0.026	1.073	1.009–1.141
**** **Factors independently associated with counts of granulocyte**			
CRP	0.005	1.022	1.007–1.037
PIP	0.012	0.785	0.650–0.949

*PCIS, pediatric critical illness score*.

## Discussion

Patients with hematological tumors are significantly more likely to develop ARDS, and these patients often have a poor prognosis (5, 9–11). This study collected data from children with hematological tumors and ARDS in our hospital in the past 5 years, and grouped them according to the prognosis of the children, the cause of ARDS, and the absolute counts of peripheral neutrophils. By analyzing the clinical characteristics of the overall and each subgroup, explore the combination of hematological tumors in children.

The prevalence of adult hematological tumors with ARDS is 5% (5). At present, there is still a lack of relevant data on children. A number of studies have shown that the prevalence of ARDS among hospitalized children is 1–4% (12, 13). Our research shows that the prevalence of ARDS in children with hematological tumors is 1.61%. In terms of age composition, most children aged 2–6 years and only two children were younger than 6 months; this may be related to the low incidence of infant hematological tumors. The main cause of ARDS is pneumonia, followed by sepsis (4, 14, 15). In this study, 75% of children with ARDS were caused by lung infections.

Seong et al. (16) found that the mortality rate of adult ARDS patients with acute leukemia was significantly higher than that of lymphoma patients. We have not found a correlation between the primary hematological tumor type and mortality in children with ARDS. Subgroup analysis showed that there was no significant difference in total hospital stay and PICU stay between children with pulmonary ARDS and children with exogenous pulmonary ARDS, and there was no significant difference in mortality, which is consistent with the results of previous studies (17–19). However, there are also reports that the mortality of exogenous pulmonary ARDS is higher than that of pulmonary ARDS, and the mortality of ARDS caused by sepsis is significantly higher than that of ARDS caused by pneumonia (13, 20). This indicates that the pathophysiological process and clinical outcome of ARDS caused by various reasons are quite different, and further study is needed. Studies have shown that when PCIS > 66, each additional point reduces the risk of death by 20% (21), which was similar to the results of our study. Besides, the *P*-value of PCIS in the univariate analysis was < 0.001, and further multivariate analysis showed that PCIS was still significantly associated with mortality, suggesting that PCIS could identify serious cases more easily, is convenient in clinical practice, and provides a certain guiding value for the prognosis of ARDS ch ildren, so as to implement timely and targeted therapeutic intervention to these children.

In our study, the mechanical ventilation time of the survival group was longer, which may be related to the extremely critical condition of the children in the non-survival group; that is, when they were admitted to the PICU, the disease progressed rapidly and missed the optimal and timely treatment. Ben-Abraham et al. (3), Wong et al. (22), Raj et al. (23) showed that the OI ratio, PIP, and PEEP could predict the clinical outcome of children with malignant tumors complicated with ARDS. However, with the exception of the increased PIP in the non-neutropenic group, we failed to find differences in OI, PIP, and PEEP in different subgroups. Possible reasons include the small sample size and differences in basic characteristics of children. The setting of PEEP value between groups is conservative and the real-time data of continuous monitoring are not used, which leads to the lag in data update and lack of representativeness.

McIntosh et al. (24) found that the VIS score within 48 h after the onset of sepsis was positively correlated with the length of stay in the ICU, the duration of the ventilator, and the mortality rate. We also found that children in the non-survival group had higher VIS scores. However, in our study, the VIS level of children with non-granulocytosis exogenous lung ARDS was significantly increased, which is inconsistent with the conclusion of Mokart et al. (25) We believe that exogenous pulmonary ARDS is often derived from sepsis, and the immune function of children with agranulocytosis is extremely low; once infected, clinical symptoms are not typical, and the low positive rate of etiological detection, the large amount of drug-resistant bacteria infection, and other factors made infection difficult to control, thus spreading widely. The increased risk of circulatory failure, combined with invasive mechanical ventilation, invasive hemodynamic monitoring, and other procedures, eventually resulted in a higher demand for vasoactive drugs in these children.

Children with ARDS have alveolar capillary leakage, and alveolar and interstitial edema; long-term positive fluid balance may worsen clinical outcomes. A retrospective cohort study by van Mourik et al. (26) found that accumulated positive fluid balance within the first 7 days increased the risk of death in ARDS patients. After adjusting for risk factors such as age, underlying disease, hemoglobin, and shock state, Zhang and Chen's study (27) found that positive fluid balance in the first 8 days was associated with increased mortality in ARDS patients. Our study also confirmed that the average positive fluid balance in the first 3 and 7 days of ARDS in the non-survival group was significantly higher than that in the survival group; the average positive fluid balance in the first 3 days was an independent risk factor for predicting death. That is, positive fluid balance was independently associated with case mortality. This reminds us that fluid resuscitation should be carefully performed in clinical practice to avoid excessive positive balance in children with ARDS.

MODS is an independent risk factor for high mortality in children with ARDS (4, 28). In this study, more than 50% of the children had MODS, and compared with the children without MODS, the mortality rate of these children was higher. More noteworthy is that compared to children with pulmonary ARDS, children with exogenous pulmonary ARDS are more likely to develop MODS during the course of the disease. ARDS in such children is mostly caused by sepsis, and MODS is more likely to occur when it develops into severe sepsis or even septic shock. Therefore, it is more important to provide timely and effective adjunctive treatment for protecting the function of vital organs of patients with severe sepsis or septic shock.

The analysis of the two remaining subgroups in this study has shown varying results. In general, significantly elevated levels of CRP and PCT are closely associated with severity of infection (29); similarly, in this study, it was also found that the elevated levels of PCT and CRP in children with exogenous pulmonary ARDS were significantly higher than those in children with pulmonary ARDS, because exogenous pulmonary ARDS was mostly attributed to sepsis, and PCT level increased more obviously in sepsis caused by systematic infection. It is worth noting that, in this study, CRP and PCT levels in children with agranulocytosis were also significantly higher than those in children without agranulocytosis, suggesting that the inflammatory response of children in ARDS complicated by hematological neoplasms with agranulocytosis was stronger, and children with immunosuppression were prone to severe infection, thus leading to rapid progression of the disease process.

## Conclusion

In conclusion, MODS, positive fluid balance, high VIS, and high PCIS score were the risk factors of poor prognosis in children with hematological neoplasms complicated with ARDS. High PCIS score and mean positive fluid balance volume of the first 3 days were independently associated with PICU mortality. Exogenous pulmonary ARDS is more likely to be complicated with MODS. In addition, exogenous pulmonary ARDS and ARDS with agranulocytosis are often accompanied by more severe infection, which should not be ignored in clinical practice. Importantly, the study also has certain limitations; it was a single-center, retrospective, small sample-sized study, and there were differences in baseline characteristics and treatment methods between groups. Therefore, the clinical characteristics and risk factors of ARDS in children with hematological neoplasms still need to be further explored in a large-scale, multi-center, prospective study.

## Data Availability Statement

The raw data supporting the conclusions of this article will be made available by the authors, without undue reservation.

## Ethics Statement

The studies involving human participants were reviewed and approved by the Ethics Committee of Shanghai Children's Medical Center (SCMCIRB-W2021023). Written informed consent to participate in this study was provided by the participants' legal guardian/next of kin.

## Author Contributions

QZ, W-tH, FY, Y-jT, and B-tN: conception and design. B-tN and Y-jT: administrative support and provision of study materials or patients. QZ, W-tH, FY, HQ, Y-jT, and B-tN: collection and assembly of data, data analysis, and interpretation. All authors contributed to the article and approved the submitted version.

## Conflict of Interest

The authors declare that the research was conducted in the absence of any commercial or financial relationships that could be construed as a potential conflict of interest.

## References

[1] Thille AW Peñuelas O Lorente JA Fernández-Segoviano P Rodriguez JM Aramburu JA. Predictors of diffuse alveolar damage in patients with acute respiratory distress syndrome: a retrospective analysis of clinical autopsies. Crit Care. (2017) 21:254. 10.1186/s13054-017-1852-5 29052522 PMC5649062

[2] Heidemann SM Nair A Bulut Y Sapru A. Pathophysiology and management of acute respiratory distress syndrome in children. Pediatr Clin North Am. (2017) 64:1017–37. 10.1016/j.pcl.2017.06.004 28941533 PMC9683071

[3] Ben-Abraham R Weinbroum AA Augerten A Toren A Harel R Vardi A. Acute respiratory distress syndrome in children with malignancy-can we predict outcome? J Crit Care. (2001) 16:54–8. 10.1053/jcrc.2001.25232 11481599

[4] Türkoglu M Erdem GU Suyani E Sancar ME Yalçin MM Aygencel G. Acute respiratory distress syndrome in patients with hematological malignancies. Hematology. (2013) 18:123–30. 10.1179/1607845412Y.0000000038 23321772

[5] Azoulay E Lemiale V Mourvillier B Garrouste-Orgeas M Schwebel C Ruckly S. Management and outcomes of acute respiratory distress syndrome patients with and without comorbid conditions. Intensive Care Med. (2018) 44:1050–60. 10.1007/s00134-018-5209-6 29881987 PMC7095161

[6] Pediatric Acute Lung Injury Consensus Conference Group. Pediatric acute respiratory distress syndrome: consensus recommendations from the pediatric acute lung injury consensus conference. Pediatr Crit Care Med. (2015) 16:428–39. 10.1097/PCC.0000000000000350 25647235 PMC5253180

[7] Shen H Qu D Na W Liu S Huang S Hui Y. Comparison of the OI and PaO(2)/FiO(2) score in evaluating PARDS requiring mechanical ventilation. Pediatr Pulmonol. (2020) 56:1182–8. 10.1002/ppul.25194 33289279

[8] Gaies MG Gurney JG Yen AH Napoli ML Gajarski RJ Ohye RG. Vasoactive-inotropic score as a predictor of morbidity and mortality in infants after cardiopulmonary bypass. Pediatr Crit Care Med. (2010) 11:234–8. 10.1097/PCC.0b013e3181b806fc 19794327

[9] Escrihuela-Vidal F Laporte J Albasanz-Puig A Gudiol C. Update on the management of febrile neutropenia in hematologic patients. Rev Esp Quimioter. (2019) 32:55–8. 31475812 PMC6755372

[10] Price DR Hoffman KL Oromendia C Torres LK Schenck EJ Choi ME. Effect of neutropenic critical illness on development and prognosis of acute respiratory distress syndrome. Am J Respir Crit Care Med. (2021) 203:504–08. 10.1164/rccm.202003-0753LE 32986956 PMC7885830

[11] Wong JJ Tan HL Lee SW Chang KTE Mok YH Lee JH. Characteristics and trajectory of patients with pediatric acute respiratory distress syndrome. Pediatr Pulmonol. (2020) 55:1000–06. 10.1002/ppul.24674 32017471

[12] Wong JJ Jit M Sultana R Mok YH Yeo JG Koh J. Mortality in pediatric acute respiratory distress syndrome: a systematic review and meta-analysis. J Intensive Care Med. (2019) 34:563–71. 10.1177/0885066617705109 28460591

[13] Yu WL Lu ZJ Wang Y Shi LP Kuang FW Qian SY. The epidemiology of acute respiratory distress syndrome in pediatric intensive care units in China. Intensive Care Med. (2009) 35:136–43. 10.1007/s00134-008-1254-x 18825369

[14] Rhee CK Kang JY Kim YH Kim JW Yoon HK Kim SC. Risk factors for acute respiratory distress syndrome during neutropenia recovery in patients with hematologic malignancies. Crit Care. (2009) 13:R173. 10.1186/cc8149 19886984 PMC2811915

[15] El-Haddad H Jang H Chen W Soubani AO. Effect of ARDS severity and etiology on short-term outcomes. Respir Care. (2017) 62:1178–85. 10.4187/respcare.05403 28559467 PMC6834371

[16] Seong GM Lee Y Hong SB Lim CM Koh Y Huh JW. Prognosis of acute respiratory distress syndrome in patients with hematological malignancies. J Intensive Care Med. (2020) 35:364–70. 10.1177/0885066617753566 29343171

[17] Agarwal R Aggarwal AN Gupta D Behera D Jindal SK. Etiology and outcomes of pulmonary and extrapulmonary acute lung injury/ARDS in a respiratory ICU in North India. Chest. (2006) 130:724–9. 10.1378/chest.130.3.724 16963669

[18] Agarwal R Srinivas R Nath A Jindal SK. Is the mortality higher in the pulmonary vs. the extrapulmonary ARDS? A meta analysis. Chest. (2008) 133:1463–73. 10.1378/chest.07-2182 17989150

[19] Gan CS Wong JJ Samransamruajkit R Chuah SL Chor YK Qian S. Differences between pulmonary and extrapulmonary pediatric acute respiratory distress syndrome: a multicenter analysis. Pediatr Crit Care Med. (2018) 19:e504–13. 10.1097/PCC.0000000000001667 30036234

[20] Zhu YF Xu F Lu XL Wang Y Chen JL Chao JX. Mortality and morbidity of acute hypoxemic respiratory failure and acute respiratory distress syndrome in infants and young children. Chin Med J. (2012) 125:2265–71. 10.3760/cma.j.issn.0366-6999.2012.13.005 22882846

[21] Zhou LB Chen J Du XC Wu SY Bai ZJ Lyu HT. Value of three scoring systems in evaluating the prognosis of children with severe sepsis. Zhongguo Dang Dai Er Ke Za Zhi. (2019) 21:898–903. 10.7499/j.issn.1008-8830.2019.09.011 31506150 PMC7390254

[22] Wong JJ Loh TF Testoni D Yeo JG Mok YH Lee JH. Epidemiology of pediatric acute respiratory distress syndrome in singapore: risk factors and predictive respiratory indices for mortality. Front Pediatr. (2014) 2:78. 10.3389/fped.2014.00078 25121078 PMC4110624

[23] Raj SS Slaven JE Rigby MR. Factors associated with survival during high-frequency oscillatory ventilation in children. J Pediatr Intensive Care. (2015) 4:146–55. 10.1055/s-0035-1559824 31110864 PMC6513134

[24] McIntosh AM Tong S Deakyne SJ Davidson JA Scott HF. Validation of the vasoactive-inotropic score in pediatric sepsis. Pediatr Crit Care Med. (2017) 18:750–57. 10.1097/PCC.0000000000001191 28486385 PMC5548505

[25] Mokart D van Craenenbroeck T Lambert J Textoris J Brun JP Sannini A. Prognosis of acute respiratory distress syndrome in neutropenic cancer patients. Eur Respir J. (2012) 40:169–76. 10.1183/09031936.00150611 22135281

[26] van Mourik N Metske HA Hofstra JJ Binnekade JM Geerts BF Schultz MJ. Cumulative fluid balance predicts mortality and increases time on mechanical ventilation in ARDS patients: an observational cohort study. PLoS ONE. (2019) 14:e0224563. 10.1371/journal.pone.0224563 31665179 PMC6821102

[27] Zhang Z Chen L. The association between fluid balance and mortality in patients with ARDS was modified by serum potassium levels: a retrospective study. PeerJ. (2015) 3:e752. 10.7717/peerj.752 25699202 PMC4327251

[28] Chang JC. Acute respiratory distress syndrome as an organ phenotype of vascular microthrombotic disease: based on hemostatic theory and endothelial molecular pathogenesis. Clin Appl Thromb Hemost. (2019) 25:1076029619887437. 10.1177/1076029619887437 31775524 PMC7019416

[29] Verbakel JY Lemiengre MB De Burghgraeve T De Sutter A Aertgeerts B Bullens DMA. Point-of-care C reactive protein to identify serious infection in acutely ill children presenting to hospital: prospective cohort study. Arch Dis Child. (2018) 103:420–26. 10.1136/archdischild-2016-312384 29269559

